# Patients With Primary Aldosteronism Respond to Unilateral Adrenalectomy With Long-Term Reduction in Salt Intake

**DOI:** 10.1210/clinem/dgz051

**Published:** 2019-11-08

**Authors:** Christian Adolf, Daniel A Heinrich, Finn Holler, Benjamin Lechner, Nina Nirschl, Lisa Sturm, Veronika Görge, Anna Riester, Tracy A Williams, Marcus Treitl, Roland Ladurner, Felix Beuschlein, Martin Reincke

**Affiliations:** 1 Medizinische Klinik und Poliklinik IV, Klinikum der Universität München, LMU München, Ziemssenstraße, Munich, Germany; 2 Divis ion of Internal Medicine and Hypertension, Department of Medical Sciences, University of Turin, Turin, Italy; 3 Klinik und Poliklinik für Radiologie, Klinikum der Universität München, LMU München, Munich, Germany; 4 Klinik für Viszeral- und Endokrine Chirurgie, Klinikum der Universität München, LMU München, Munich, Germany; 5 Klinik für Endokrinologie, Diabetologie und Klinische Ernährung, Universitätsspital Zürich, Zurich, Switzerland

**Keywords:** primary aldosteronism, sodium excretion, salt intake, hypertension, cardiovascular risk, adrenalectomy

## Abstract

**Context:**

High dietary salt intake is known to aggravate arterial hypertension. This effect could be of particular relevance in the setting of primary aldosteronism (PA), which is associated with cardiovascular damage independent of blood pressure levels. The aim of this study was to determine the impact of therapy on salt intake in PA patients.

**Patients and Methods:**

A total of 148 consecutive PA patients (66 with unilateral and 82 with bilateral PA) from the database of the German Conn’s Registry were included. Salt intake was quantified by 24-hour urinary sodium excretion before and after initiation of PA treatment.

**Study design:**

Observational longitudinal cohort study.

**Setting:**

Tertiary care hospital.

**Results:**

At baseline, unilateral PA patients had a significantly higher urinary sodium excretion than patients with bilateral disease (205 vs 178 mmol/d, *P* = 0.047). Higher urinary sodium excretion correlated with an increased cardiovascular risk profile including proteinuria, impaired lipid, and glucose metabolism and was associated with higher daily doses of antihypertensive drugs to achieve blood pressure control. In unilateral disease, urinary sodium excretion dropped spontaneously to 176 mmol/d (*P* = 0.012) 1 year after unilateral adrenalectomy and remained low at 3 years of follow-up (174 mmol/d). In contrast, treatment with mineralocorticoid receptor antagonists (MRA) in bilateral PA patients was not associated with a significant change in urinary sodium excretion at follow-up (179 mmol/d vs 183 mmol/d).

**Conclusion:**

PA patients consuming a high-salt diet, estimated based on urinary sodium excretion, respond to adrenalectomy with a significant reduction of salt intake, in contrast to MRA treatment.

The triumph of sodium chloride (salt) began in the age of ancient Babylonia and Egypt, where salt was already used in the preservation of food. As a symbol of the importance of salt, Roman soldiers received their salary, derived from the Latin word *salarium* (salt), in part as salt itself. Nowadays, salt remains an important spice and preservative and very popular especially as part of Western diet. However, high salt intake has undesirable health effects and is regarded as an independent cardiovascular risk factor. Beside its negative impact on left ventricular mass and arterial stiffness, high salt intake results in an elevated risk of stroke and cardiovascular disease as well as an increase of blood pressure (1–4). For this reason, renunciation of salt intake is a common and effective public health approach of lowering blood pressure, with an even more distinct impact in resistant hypertension (5–8).

One of the main regulators of salt and water balance is the steroid hormone aldosterone, which is synthesized in the zona glomerulosa of the adrenal cortex and is stimulated by increased renin and angiotensin II plasma levels. Aldosterone acts predominantly via the epithelial sodium channel (ENaC) in the distal nephron leading to increased sodium reabsorption and loss of potassium. Primary aldosteronism (PA) is characterized by excessive secretion of aldosterone despite suppressed renin levels. It affects 5% to 10% of patients with high blood pressure and is the most frequent cause of endocrine hypertension (9, 10). Aldosterone excess in PA leads to hypervolemia by sodium and water retention and causes target organ damage through pro-inflammatory and pro-fibrotic effects, even independent of blood pressure changes (11, 12).

A recent study showed that reducing dietary salt intake in PA patients results in a substantial reduction of left ventricular mass index and therefore is of clinical relevance (13). Although many experimental studies have indicated that inadequately high salt intake together with aldosterone excess is deleterious for target organ damage, data about salt intake especially after initiation of treatment in PA patients are very limited (13, 14).

It was the objective of this study to investigate long-term intake of salt in a large cohort of PA patients and to analyze its association with treatment—unilateral adrenalectomy or mineralocorticoid receptor blockade by spironolactone. It was our hypothesis that PA is associated with high sodium consumption and that remission from excessive aldosterone levels and action (mineralocorticoid receptor antagonist [MRA] treatment) might positively affect salt intake.

## Methods

From 2008 to 2015, we prospectively enrolled 323 patients with PA in the Munich center of the German Conn’s Registry. For the present study, we selected patients fulfilling the following criteria: confirmed PA treated by either adrenalectomy (ADX) in unilateral PA or MRA in bilateral PA and 24-hour urinary sodium excretion measurement at baseline, and 1 and 3 years after initiation of specific PA directed treatment, respectively. Patients with urine volume <500 mL/d were excluded to ensure completeness of 24-hour urine collection. We identified 148 patients fulfilling the inclusion criteria; they became our study cohort. All patients gave written informed consent, and the protocol of the German Conn’s Registry was approved by the ethics committee of the University of Munich.

At time of diagnosis and at each visit, patients underwent standard procedures including collection of anthropometric data, clinical characteristics, current medication, and laboratory testing. Blood pressure was measured using an automated device over 24 hours. To estimate daily salt intake, the patients conducted a 24-hour urine collection at baseline after adjustment of medication for further testing of PA to determine urinary sodium excretion at each visit.

For diagnosis of PA, patients underwent standardised testing, which was performed according to Endocrine Society Practice Guidelines (10). The diagnosis of PA was confirmed by an elevated plasma aldosterone to renin ratio (cutoff 12.0 ng/U, sitting position) followed by an abnormal confirmatory test (ie, salt loading test, captopril challenge test, or both). Computed tomography scanning in combination with adrenal vein sampling was used for subtype diagnosis, as described elsewhere (15). In 7.4% of the patients, blood pressure medication was stopped, whereas in the remaining patients, alpha 1-adrenergic receptor (doxazosin) or calcium-channel blockers (verapamil) replaced medication. ADX was offered to all patients with unilateral PA. Patients with unilateral PA who did not undergo ADX were not included in the study. All patients with bilateral PA were treated with MRAs using spironolactone with a starting dose of 25 to 50 mg/d in the majority of cases. Reevaluation at follow-up followed a standardized protocol.

Genotyping for KCNJ5, ATP1A1, ATP2B3, and CACNA1D was performed in surgically resected tumor tissue of unilateral PA as described elsewhere (16).

### Statistical analysis

All values are expressed as median, 25th and 75th percentile, if not mentioned otherwise. Body mass index (BMI) was calculated as weight in kilograms divided by the square of the height in meters. Data between groups were compared using Mann-Whitney *U* test or Kruskal-Wallis test, respectively. Within-group changes from baseline to follow-up were calculated by Wilcoxon signed-rank test. Spearman’s rank order was used to perform bivariate correlation analysis. Stepwise multiple regression analysis was performed for multivariate analysis. Two-tailed probability values <5% were considered to be statistically significant. Statistical analysis was done using standard statistical software (SPSS 25, IBM, Chicago, IL).

## Results

Clinical and biochemical characteristics of all patients are summarized in Table 1. In total, 38% of patients (n = 56) were female. Patients had a median age of 51 years, were overweight with a BMI of 27.4 kg/m^2^, and had low potassium and high aldosterone levels, as expected. Twenty-four-hour systolic blood pressure (SBP) and diastolic blood pressure (DBP) was elevated with 144/93 mm Hg despite receiving a median of 2.5 doses of antihypertensive drugs per day (DDD). Median urinary sodium excretion was 184 mmol/d, reflecting a daily salt consumption of more than 10 g, which is more than twice of the amount recommended by the World Health Organization (17).

**Table 1.** Baseline, 1-, and 3-Year Follow-up Characteristics of All Patients With Primary Aldosteronism

Patient Characteristics (n = 148)	No.	Baseline	After 1 Y	*P*	After 3 Y	*P*
Sex, F/M	148	56/92	--	NC	--	NC
Age, y	148	51 [45; 59]	--	NC	--	NC
BMI, kg/m^2^	148	27.4 [24.3; 31.2]	27.4 [24.0; 30.5]	0.332	27.8 [24.5; 30.5]	0.339
Aldosterone, ng/L	148	170 [107; 263]	129 [59; 239]	0.109	155 [83; 279]	0.986
Plasma renin, mU/L	148	4.1 [2.1; 8.5]	16.0 [6.6; 28.1]	**<0.001**	19.6 [7.2; 39.9]	**<0.001**
SBP, mm Hg	148	150 [137; 166]	133 [123; 143]	**<0.001**	131 [121; 141]	**<0.001**
DBP, mm Hg	148	93 [84; 102]	87 [80; 93]	**<0.001**	86 [79; 93]	**<0.001**
24-h SBP, mm Hg	114	144 [137; 154]	132 [123; 139]	**<0.001**	130 [121; 138]	**<0.001**
24-h DBP, mm Hg	114	93 [83; 99]	82 [76; 87]	**<0.001**	82 [77; 87]	**<0.001**
DDD, n	148	2.5 [1.0; 4.0]	1.7 [0.5; 3.6]	**0.004**	1.7 [0.5; 3.0]	**<0.001**
Serum sodium, mmol/L	148	141 [139; 142]	139 [137; 140]	**<0.001**	140 [138; 141]	**0.001**
Serum potassium, mmol/L	148	3.5 [3.2; 3.8]	4.1 [3.9; 4.4]	**<0.001**	4.4 [4.1; 4.6]	**<0.001**
Serum creatinine, mg/dL	148	0.9 [0.7; 1.0]	1.0 [0.8; 1.2]	**<0.001**	1.0 [0.9; 1.2]	**<0.001**
GFR, mL/min/1.73 m^2^	148	85 [72; 100]	73 [59; 84]	**<0.001**	69 [58; 81]	**<0.001**
HDL-C, mg/dL	148	56 [45; 69]	50 [41; 64]	**<0.001**	53 [44; 64]	**<0.001**
LDL-C, mg/dL	148	120 [98; 148]	121 [95; 143]	0.448	119 [85; 143]	0.603
Triglycerides, mg/dL	148	95 [67; 135]	119 [82; 175]	**<0.001**	120 [83; 177]	**<0.001**
Total cholesterol, mg/dL	148	193 [173; 221]	191 [168; 223]	0.807	191 [163; 224]	0.859
FPG, mg/dL	148	98 [91; 110]	99 [91; 106]	0.139	99 [92; 109]	0.197
HbA1c, %	130	5.3 [5.1; 5.7]	5.5 [5.2; 5.8]	**<0.001**	5.4 [5.2; 5.8]	**<0.001**
proBNP, pg/mL	137	86 [52; 185]	52 [29; 93]	**<0.001**	47 [26; 120]	**<0.001**
Proteinuria, mg/d	148	143 [109; 210]	104 [83; 126]	**<0.001**	112 [85; 134]	**<0.001**
24-h urinary potassium, mmol/d	148	87 [67; 125]	68 [52; 87]	**<0.001**	69 [49; 84]	**<0.001**
24-h urinary sodium, mmol/d	148	184 [146; 253]	177 [128; 238]	**0.027**	182 [136; 240]	0.126
Estimated salt intake, g/d	148	10.8 [8.5; 14.8]	10.4 [7.5; 13.9]	**0.027**	10.6 [8.0; 14.0]	0.126

Data are given as median, and 25th and 75th percentile in square brackets. Significance is marked in bold. Comparisons to baseline values were performed by Wilcoxon signed-rank test.

Abbreviations: 24-h DBP, 24-hour diastolic blood pressure; 24-h SBP, 24-hour systolic blood pressure; DBP, diastolic blood pressure; DDD, defined daily doses of antihypertensive medication; FPG, fasting plasma glucose; GFR, glomerular filtration rate; HDL-C, high-density lipoprotein cholesterol; LDL-C, low-density lipoprotein cholesterol; NC, not calculated; proBNP, pro b-type natriuretic peptide; SBP, systolic blood pressure.

There was no significant difference according to age, sex, BMI, or blood pressure in bilateral versus unilateral PA (Table 2). Unilateral PA patients had lower potassium (*P* < 0.001) and higher plasma aldosterone values (*P* < 0.001) as well as higher 24-hour urinary potassium excretion (105 mmol/d vs 80 mmol/d; *P* < 0.001), as expected. Patients with unilateral PA had higher pro b-type natriuretic peptide (110 pg/mL vs 78 pg/mL; *P* = 0.013) and urinary sodium excretion (205 mmol/d vs 178 mmol/d; *P* = 0.047).

**Table 2.** Baseline, 1-, and 3-Year Follow-up Characteristics of Patients With Primary Aldosteronism According to Subtype

Patient Characteristics	Unilateral Primary Aldosteronism (n = 66)			*P*	Bilateral Primary Aldosteronism (n = 82)			*P*
Time of Assessment	Baseline	1 y After ADX	3 y After ADX		Baseline	1 y After MRA	3 y After MRA	
Age, y	52 [46; 59]	--	--	NC	51 [44; 59]	--	--	NC
Sex, F/M	24/42	--	--	NC	32/50	--	--	NC
BMI, kg/m^2^	28.2 [24.9; 32.1]	28.4 [24.5; 31.2] --	-- 28.2 [24.7; 30.3]	0.182 0.805	27.0 [23.9; 30.7]	26.4 [23.6; 30.1] --	-- 27.4 [23.8; 31.1]	0.948 0.108
Aldosterone, ng/L	226 [153; 368]^a^	57 [35; 92] --	-- 80 [51; 111]	**<0.001** **<0.001**	134 [100; 192]^a^	208 [139; 315] --	-- 250 [167; 354]	**<0.001** **<0.001**
Plasma renin, mU/L	4.0 [2.0; 9.5]	16.4 [8.0; 27.8] --	-- 21.0 [8.0; 40.4]	**<0.001** **<0.001**	4.1 [2.7; 7.4]	15.4 [5.5; 28.8] --	-- 19.4 [7.1; 39.2]	**<0.001** **<0.001**
SBP, mm Hg	152 [139; 166]	135 [122; 147] --	-- 132 [123; 139]	**<0.001** **<0.001**	149 [137; 166]	132 [124; 141] --	-- 130 [119; 143]	**<0.001** **<0.001**
DBP, mm Hg	94 [84; 102]	89 [82; 95] --	-- 86 [80; 93]	**0.001** **0.001**	93 [85; 101]	85 [79; 92] --	-- 86 [78; 93]	**<0.001** **<0.001**
24-h SBP, mm Hg^b^	145 [139; 154]	131 [124; 138] --	-- 131 [119; 137]	**<0.001** **<0.001**	143 [134; 155]	132 [122; 140] --	-- 129 [124; 139]	**<0.001** **<0.001**
24-h DBP, mm Hg^b^	93 [84; 99]	81 [76; 87] --	-- 81 [77; 87]	**<0.001** **<0.001**	90 [83; 99]	82 [77; 87] --	-- 82 [77; 87]	**<0.001** **<0.001**
DDD, no.	3.0 [1.4; 4.0]	1.2 [0.0; 3.4] --	-- 1.0 [0.0; 2.7]	**<0.001** **<0.001**	2.0 [1.0; 4.3]	2.0 [1.0; 3.7] --	-- 2.0 [0.7; 3.2]	0.759 0.712
Serum sodium, mmol/L	141 [139; 143]^a^	139 [138; 141] --	-- 140 [138; 141]	**<0.001** **0.008**	140 [139; 142]^a^	139 [137; 140] --	-- 139 [138; 141]	**<0.000** **0.043**
Serum potassium, mmol/L	3.4 [3.0; 3.5]^a^	4.2 [3.9; 4.5] --	-- 4.4 [4.2; 4.5]	**<0.001** **<0.001**	3.7 [3.4; 3.9]^a^	4.1 [3.9; 4.3] --	-- 4.4 [4.1; 4.6]	**<0.001** **<0.001**
Serum creatinine, mg/dL	0.9 [0.7; 1.1]	1.1 [0.8;1.2] --	-- 1.0 [0.9; 1.2]	**<0.001** **<0.001**	0.9 [0.7; 1.0]	1.0 [0.8; 1.1] --	-- 1.0 [0.9; 1.1]	**<0.001** **<0.001**
GFR, mL/min/1.73 m^2^	84 [69; 100]	69 [57; 81] --	-- 65 [54; 76]	**<0.001** **<0.001**	85 [75; 99]	76 [63; 87] --	-- 73 [59; 83]	**<0.001** **<0.001**
HDL-C, mg/dL	56 [45; 66]	49 [42; 64] --	-- 53 [42; 62]	**<0.001** **0.015**	59 [45; 71]	53 [40; 64] --	-- 53 [45; 66]	**<0.001** **0.012**
LDL-C, mg/dL	116 [93; 150]	114 [92; 143] --	-- 108 [83; 138]	0.969 0.291	122 [101; 144]	124 [101; 143] --	-- 123 [87; 149]	0.189 0.792
Triglycerides, mg/dL	86 [65; 128]^a^	109 [79; 174] --	-- 119 [85; 166]	**<0.001** **<0.001**	108 [71; 142]^a^	128 [87; 178] --	-- 120 [83; 188]	**<0.001** **0.001**
Total cholesterol, mg/dL	187 [168; 224]	187 [163; 217] --	-- 185 [157; 219]	0.857 0.673	196 [175; 220]	196 [173; 231] --	-- 195 [170; 227]	0.693 0.526
FPG, mg/dL	99 [92; 110]	97 [91; 106] --	-- 98 [92; 109]	**0.044** 0.763	98 [90; 113]	99 [91; 109] --	-- 100 [93; 111]	0.848 **0.047**
HbA1c, %^b^	5.3 [5.0; 5.7]	5.5 [5.2; 5.7] --	-- 5.4 [5.2; 5.7]	**0.002** **0.001**	5.4 [5.1; 5.7]	5.5 [5.3; 5.8] --	-- 5.5 [5.3; 5.9]	**<0.001** **<0.001**
proBNP, pg/mL^b^	110 [62; 212]^a^	52 [34; 86] --	-- 48 [31; 126]	**<0.001** **<0.001**	78 [43; 135]^a^	51 [28; 115] --	-- 47 [23; 93]	**<0.001** **<0.001**
Proteinuria, mg/d	176 [130; 254]^a^	98 [78; 122] --	-- 103 [80; 125]	**<0.001** **<0.001**	128 [103; 160]^a^	106 [84; 129] --	-- 115 [88; 144]	**<0.001** **0.005**
24-h urinary potassium, mmol/d	105 [76; 143]^a^	67 [51; 86] --	-- 70 [48; 82]	**<0.001** **<0.001**	80 [62; 101]^a^	70 [53; 87] --	-- 67 [50; 87]	**0.001** **0.001**
24-h urinary sodium, mmol/d	205 [161; 263]^a^	176 [128; 256] --	-- 174 [134; 226]	**0.012** **0.007**	178 [132; 222]^a^	179 [117; 235] --	-- 183 [138; 247]	0.475 0.584
Estimated salt intake, g/d	11.9 [9.4; 15.4]^a^	10.3 [7.5; 15.0] --	-- 10.2 [7.8; 13.2]	**0.012** **0.007**	10.4 [7.7; 12.9]^a^	10.4 [6.8; 13.7] --	-- 10.7 [8.1; 14.4]	0.475 0.584

Data are given as median, and 25th and 75th percentile in square brackets. Significance is marked in bold. Comparisons to baseline values were performed by Wilcoxon signed-rank test and by Mann-Whitney *U* test.

Abbreviations: 24-h DBP, 24-hour diastolic blood pressure; 24-h SBP, 24-hour systolic blood pressure; ADX, adrenalectomy; BPA, bilateral primary aldosteronism; DBP, diastolic blood pressure; DDD, defined daily doses of antihypertensive medication; FPG, fasting plasma glucose; GFR, glomerular filtration rate; HbA1c, hemoglobin A1c; HDL-C, high-density lipoprotein cholesterol; LDL-C, low-density lipoprotein cholesterol; MRA, mineralocorticoid receptor antagonist treatment; NC, not calculated; proBNP, pro b-type natriuretic peptide; SBP, systolic blood pressure; UPA, unilateral primary aldosteronism.

^a^Differences between baseline values of both groups, for *P* < 0.05.

^b^Because of incomplete data, the calculations for 24-h SBP and 24-h DBP (UPA n = 52, BPA n = 61), HbA1c (UPA n = 55, BPA n = 75), and pro-BNP (UPA n = 59, BPA n = 78) were performed with a reduced number of patients as listed in brackets.

**Figure 1.**
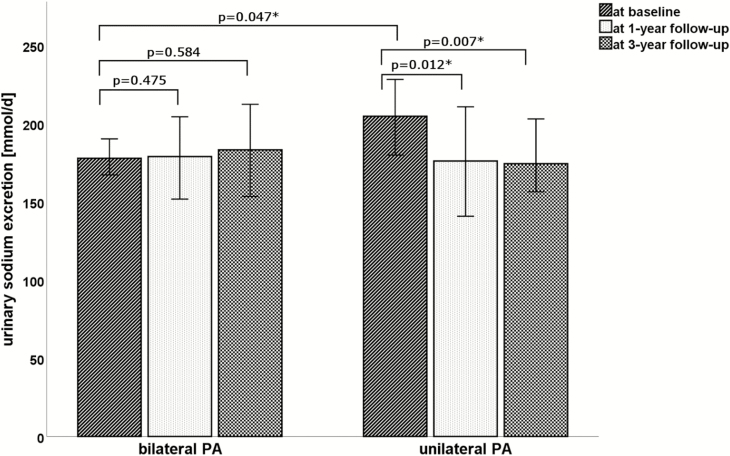
Twenty-four-hour urinary sodium excretion at baseline and at 1- and 3-year follow-up in unilateral and bilateral primary aldosteronism.Median and 95% confidence interval are shown. *Significance. Abbreviation: PA, primary aldosteronism.

Patients were treated by ADX and MRA according to adrenal vein sampling results, respectively, and underwent reassessment of salt intake 1 year after start of treatment. Serum potassium levels normalized in both subgroups and urinary potassium excretion, blood pressure, proteinuria, and pro b-type natriuretic peptide were significantly reduced (Tables 1 and 2). In patients with unilateral PA, there was a significant decrease in aldosterone levels and urinary sodium excretion (*P* < 0.001; *P* = 0.012) after ADX, which was not the case in patients with bilateral disease treated with MRA. This drop was maintained at 3 years in unilateral PA, whereas sodium excretion remained high in bilateral disease (Fig. 1). Despite these changes, 95% of patients (n = 141) remained at an estimated salt intake above the recommended limit.

**Figure 2.**
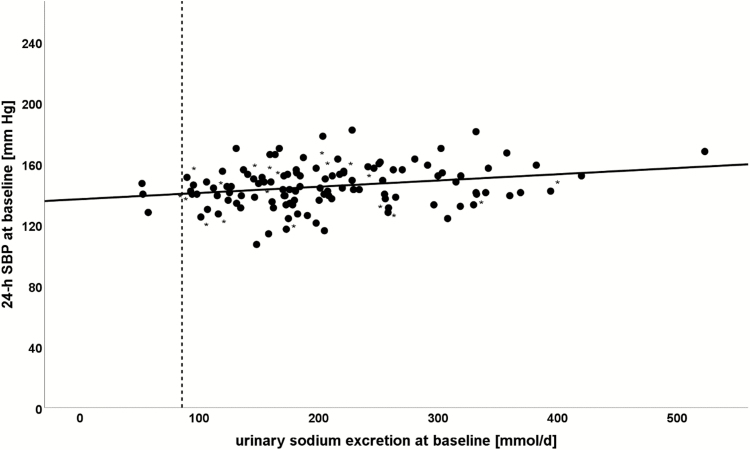
Correlation of 24-hour systolic blood pressure with 24-hour urinary sodium excretion at baseline.*Patients carrying KCNJ5 mutation. The dashed line marks an estimated salt intake of 5 g/d as recommended by the World Health Organization. Abbreviation: 24-h SBP, 24-hour systolic blood pressure.

Higher sodium excretion at baseline was predominantly found in males and correlated with features of the metabolic syndrome including overweight, large waist circumference, and dyslipidemia. In addition, higher sodium excretion was accompanied by higher 24-hour SBP and 24-hour DBP despite intake of a higher number of antihypertensive drugs (Fig. 2 and Table 3).

**Table 3.** Univariate Analyses of the Associations Between 24-Hour Sodium Excretion and Parameters of Metabolism and Blood Pressure in All Patients With Primary Aldosteronism

Parameters at Visit	Male	BMI	Proteinuria	HDL-C	LDL-C	Triglycerides	HbA1c	FPG	24-h SBP	DDD
24-h urinary sodium at baseline, mmol/d	**<0.001**	**<0.001**	**0.001**	**0.001**	0.088	**0.044**	0.542	0.253	**0.013**	**0.028**
24-h urinary sodium at 1-y follow-up, mmol/d	**<0.001**	**<0.001**	**0.001**	**<0.001**	0.338	**<0.001**	**0.013**	**0.011**	0.057	**0.009**
24-h urinary sodium at 3-year follow-up, mmol/d	**<0.001**	**<0.001**	**<0.001**	**0.001**	0.917	**0.002**	**0.035**	**0.004**	0.198	**<0.001**

Data are given as *P* values. Significance is marked in bold. Correlation analysis was performed using Spearman’s rank-order test.

Abbreviations: 24-h SBP, 24-hour systolic blood pressure; DDD, defined daily doses of antihypertensive medication; FPG, fasting plasma glucose; HDL-C, high-density lipoprotein cholesterol; LDL-C, low-density lipoprotein cholesterol.

Moreover, we detected significantly lower sodium excretion in APA patients showing KCNJ5 mutation compared with patients with a wild-type genotype in baseline univariate analysis (161 mmol/d vs 228 mmol/d; *P* = 0.008). After adjustment for sex, because KCNJ5 mutation is found more frequently in females (in our study, 73% females), these differences disappeared, in line with a sex-related influence of salt intake rather than a specific genetic impact of the KCNJ5 mutation (Fig. 2 and Table 3).

**Figure 3.**
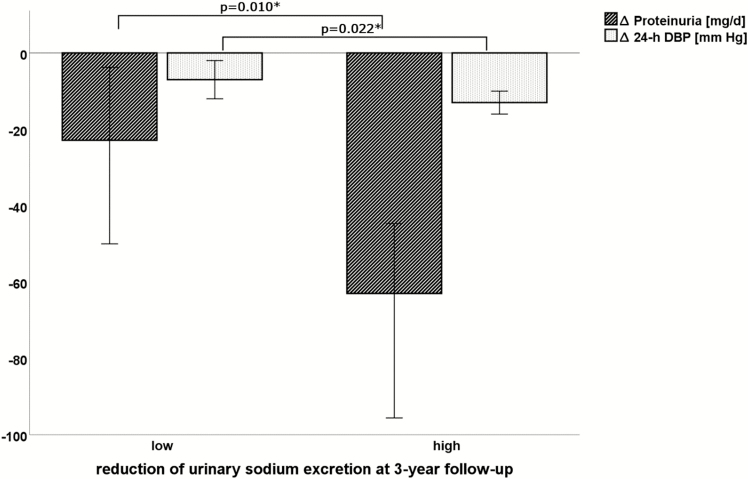
Changes in 24-hour diastolic blood pressure and proteinuria at 3-year follow-up according to high or low change in 24-hour urinary sodium excretion compared with baseline.Median and 95% confidence interval are shown. *Significance. Abbreviations: 24-h DBP, 24-hour diastolic blood pressure; Δ 24-h DBP, 24-h DBP at 3-year follow-up—24-h DBP at baseline; Δ Proteinuria, proteinuria at 3-year follow-up—proteinuria at baseline.

Proteinuria was another factor closely linked with high salt intake both at baseline and at after-care visits (Table 3). In this context, a reduction of urinary sodium excretion after initiation of treatment was accompanied by a decline in proteinuria at 1- (*r* = 0.278; *P* = 0.001) as well as 3-year (*r* = 0.242; *P* = 0.003) follow-up (Fig. 3).

After initiation of treatment of PA urinary sodium excretion still correlated with parameters of metabolic syndrome including dyslipidemia, higher fasting plasma glucose, hemoglobin A1c, and BMI (Table 3). Similarly, DDDs were strongly correlated with urinary sodium excretion both at 1- (*r* = 0.213; *P* = 0.009) and 3-year reassessment (*r* = 0.379; *P* < 0.001).

Over time, we recorded 15 cardiovascular events in our patients. Dividing the total cohort symmetrically into low and high urinary sodium excretion according to baseline values, we observed 10 cardiovascular events in the high sodium group, but only 5 in the low sodium group (*P* = NS). Taking into account the average sodium excretion from baseline to 3-year follow-up, 11 of 15 cardiovascular events occurred in the high-sodium group.

## Discussion

PA is attracting attention as the most frequent form of endocrine hypertension. High aldosterone levels per se are not regarded as a cardiovascular risk factor, as seen in indigenous people in New Guinea with chronic salt deficiency and consecutive secondary aldosteronism (18). However, in PA aldosterone levels are elevated inappropriately for salt status, resulting in target organ damage independent of blood pressure levels. Potential mechanisms involved are detailed in a recent report by Funder (19).

To our knowledge, this is the first study to evaluate spontaneous salt intake, as estimated by urinary sodium excretion, in long-term follow-up in unilateral and bilateral PA patients. At baseline, urinary sodium excretion in both subgroups was much higher than recommended by the World Health Organization and higher than in population-based studies in Germany. Assuming that the sodium excreted in urine arose from diet, estimated median daily salt intake was 11.9 g in men and 9.4 g in women, which tends to be slightly higher than the German median of 10.0 g/d in men and 8.4 g/d in women (20). Thereby 64% of our PA patients had a salt intake of more than 10 g/d (75% of men and 55% of women).

Salt intake itself is a well-known risk factor for hypertension but also for cardiovascular disease (1–4, 21–23). In a Finnish study, it has been shown that an increase of daily salt intake of 100 mmol (~5.8 g salt) is associated with an increase of cardiovascular events of 45% over a 7-year follow-up and predicted mortality in overweight men (24). High sodium intake is associated with higher risk for stroke, independent of blood pressure changes (25). Moreover, salt restriction has been demonstrated to improve blood pressure–lowering effects of antihypertensive drugs (26). In patients with arterial hypertension or metabolic syndrome blood pressure–lowering effects of low-salt diet are even more distinct than in normotensives, with a decrease of 23 mm Hg in systolic and 9 mm Hg in diastolic blood pressure via low-salt diet in patients with resistant hypertension (5, 27). The reduction of salt intake might therefore be as beneficial as reduction of body weight or smoking cessation for cardiovascular risk (28). In line with these findings, Pimenta et al found a correlation between the amount of salt intake and the severity of obstructive sleep apnea in PA, whereas Takakuwa et al reported of improved nocturnal blood pressure levels following dietary sodium restriction (29, 30).

In line with findings from patients with essential hypertension, higher sodium intake was associated with significantly higher DDD for blood pressure control in both unilateral and bilateral PA patients. Additionally, there was a positive correlation between the excretion of urinary sodium and proteinuria at baseline and at follow-up. Decline in urinary sodium excretion at follow-up was associated with a decline in proteinuria in univariate analysis. Proteinuria itself can be an early sign of renal damage representing both organ damage of aldosterone excess in PA and as well as an independent cardiovascular risk factor (31, 32). Our findings are in line with other studies, suggesting an impact of high-salt diet on cardiovascular risk in PA even after specific treatment (33, 34).

Estimated salt intake at baseline was higher in unilateral than in bilateral PA patients (11.9 g/d vs 10.4 g/d). Following ADX, estimated daily salt intake was reduced from 11.9 g to 10.2 g without any further lifestyle intervention in the unilateral group but remained unchanged in the bilateral subgroup treated with MRA. In conjunction with the findings of He et al (35), who reported a significant decrease in cardiovascular events by 20% caused by a single reduction of salt intake of about 2 g/d, the drop of 1.8 g/d (15%) in the unilateral PA patients is very likely of clinical relevance. Catena et al. reported significantly greater reduction of left ventricular mass index in patients with reduction of urinary sodium excretion after treatment of PA (13). In combination with the negative impact of increased left ventricular hypertrophy on cardiovascular risk, this further supports our hypothesis.

In addition to the sodium-retaining function of aldosterone, several physiological pathways have been proposed by which aldosterone affects sodium intake. These include sodium sensing via the ENaC in the tongue and salt appetite regulation in the brain. The ENaC is expressed in the gustatory system and more precisely in the taste buds of the tongue. Although not all mechanisms are completely understood, treatment with amiloride is known to reduce taste intensity for sodium (36). In rodent studies, mice with ENaC-alpha knockdown in the tongue showed almost complete loss of salt attraction in contrast to water (37). Pretreatment with high doses of deoxycorticosterone, a potent mineralocorticoid, caused an increase in saline preference even in ranges, which seemed uneatable for untreated rats (38, 39). Sakamoto et al. reported lower amiloride-sensitive salt taste nerve responses in aldosterone/sodium chloride treated rats, which could explain the increase in saline preference and consecutively the rise in salt intake (40).

The most popular hypothesis based on evidence from rodent studies is that aldosterone is involved in salt appetite via activation of mineralocorticoid receptor in the brain and up-regulation of serum- and glucocorticoid-induced kinase SGK1. Rats injected with aldosterone into the cerebral fourth ventricle or the amygdala increased daily salt intake, an effect that could be blocked by pretreatment with intracerebroventricular application of MRA including spironolactone (41, 42). The exact mechanisms still remain uncertain but it was interesting to note that salt appetite could not be blocked by peripheral application of MRA (43). These findings are in accordance with our results and could explain why there was no change in salt intake observed in bilateral PA patients after treatment with spironolactone.

In contrast to our findings, Catena et al found a significant decrease of urinary sodium excretion not only in a cohort of 30 patients with unilateral PA undergoing ADX, but also in 35 bilateral PA patients after 1 year of MRA treatment (13). The main difference to their study protocol was the higher starting dosage of 50 to 100 mg/d for MRA treatment compared with low-dose treatment with a starting dose of 25 to 50 mg/d, in accordance with Endocrine Society Practice Guidelines (10), in the current study. In our patients, dosage escalation was mostly limited by side effects including gynecomastia. Dosage at follow-up was a median of 50 mg/d in our cohort, contrasted by a dosage between 50 and 250 mg/d in the Italian group. Unlike Catena et al., who reinforced their advice to reduce sodium intake in close intervals up to the final 1-year follow-up, in our center, follow-up visits and nutritional counseling were less frequent. In this context, insufficient blockade of mineralocorticoid receptors by low-dose MRA treatment has to be considered as well as effective lifestyle intervention by the Italian group that may explain the controversial results. In summary, PA treatment by ADX seems to be more effective in the sustained reduction of salt intake and for this reason could be favorable concerning blood pressure control and cardiovascular risk.

Our study represents a retrospective analysis of prospectively collected data of patients included in our Munich center of the German Conn’s Registry. A limitation in this context is the lack of dietary assessments (dietary recall or food frequency questionnaire) to evaluate sodium intake. However, dietary assessments often deal with difficulties quantifying sodium concentration in different sources as well as underestimation in case of social acceptability (44). A minor limitation could be the moderate number of patients fulfilling our strict inclusion criteria.

The strengths of our study include the prospective standardized collection of all data and biomaterial within the context of the German Conn’s Registry. This allowed us to include a large number of patients both with unilateral and bilateral disease who were adequately phenotyped in a standardized fashion and had a 3-year follow-up. Furthermore, we used 24-hour urinary sodium excretion to estimate dietary salt intake, which is considered the gold standard, despite its pitfalls concerning complete 24-hour collection and variability (45).

## Abbreviations

ADX: adrenalectomy
BMI: body mass index
DBP: diastolic blood pressure
DDD: doses of antihypertensive drugs per day
ENaC: epithelial sodium channel
MRA: mineralocorticoid receptor antagonist
PA: primary aldosteronism
SBP: systolic blood pressure.

## Additional Information

**Disclosure Summary:** The authors have nothing to disclose.

***Data Availability:*** All data generated or analyzed during this study are included in this published article or in the data repositories listed in References.
